# Massively Parallel Sequencing for Rare Genetic Disorders: Potential and Pitfalls

**DOI:** 10.3389/fendo.2020.628946

**Published:** 2021-02-19

**Authors:** Aideen M. McInerney-Leo, Emma L. Duncan

**Affiliations:** ^1^ Dermatology Research Centre, University of Queensland Diamantina Institute, The University of Queensland, Brisbane, QLD, Australia; ^2^ Department of Twin Research & Genetic Epidemiology, Faculty of Life Sciences and Medicine, School of Life Course Sciences, King’s College London, London, United Kingdom

**Keywords:** gene discovery, massively parallel sequencing, skeletal dysplasias, whole exome sequencing, rare genetic bone disorder

## Abstract

There have been two major eras in the history of gene discovery. The first was the era of linkage analysis, with approximately 1,300 disease-related genes identified by positional cloning by the turn of the millennium. The second era has been powered by two major breakthroughs: the publication of the human genome and the development of massively parallel sequencing (MPS). MPS has greatly accelerated disease gene identification, such that disease genes that would have taken years to map previously can now be determined in a matter of weeks. Additionally, the number of affected families needed to map a causative gene and the size of such families have fallen: *de novo* mutations, previously intractable by linkage analysis, can be identified through sequencing of the parent–child trio, and genes for recessive disease can be identified through MPS even of a single affected individual. MPS technologies include whole exome sequencing (WES), whole genome sequencing (WGS), and panel sequencing, each with their strengths. While WES has been responsible for most gene discoveries through MPS, WGS is superior in detecting copy number variants, chromosomal rearrangements, and repeat-rich regions. Panels are commonly used for diagnostic purposes as they are extremely cost-effective and generate manageable quantities of data, with no risk of unexpected findings. However, in instances of diagnostic uncertainty, it can be challenging to choose the right panel, and in these circumstances WES has a higher diagnostic yield. MPS has ethical, social, and legal implications, many of which are common to genetic testing generally but amplified due to the magnitude of data (*e.g.*, relationship misattribution, identification of variants of uncertain significance, and genetic discrimination); others are unique to WES and WGS technologies (*e.g.*, incidental or secondary findings). Nonetheless, MPS is rapidly translating into clinical practice as an extremely useful part of the clinical armamentarium.

## The Recognition of Rare Genetic Disorders

In starting this paper exploring massively parallel sequencing (MPS) technologies for rare genetic disorders with particular reference to skeletal diseases, it is extremely fitting that the first description of any monogenic disorder was black bone disease (now known as alkaptonuria). Archibald Garrod, a UK physician, commented in 1902 that the constellation of symptoms constituting alkaptonuria “was apt to make its appearance in two or more brothers and sisters” (1). Increased occurrence in siblings does not necessarily indicate a genetic disorder (increased familiality may also reflect environmental sharing); but crucially Garrod also noted that they were commonly “the offspring of marriages of first cousins who did not themselves exhibit this anomaly … and among whose forefathers there is no record of its having occurred”. Through the world-wide dissemination of Gregor Mendel’s gardening experiences (2), the modern reader would rapidly recognize this “peculiar mode of incidence….well known in connexion with some other conditions” as a classic description of a recessive monogenic disorder.

Monogenic disorders arise due to carriage of highly penetrant variants affecting a single gene. The presence or absence of disease can be predicted from the presence or absence of the variant(s) of interest. With some allowance for differential penetrance and expressivity, the mathematical and predictable inheritance patterns of monogenic disorders enable meaningful genetic counseling to affected individuals and known carriers and to parents with a child affected by a *de novo* dominant mutation. Monogenic disorders are individually rare but cumulatively affect 1% of the worldwide population (3) and include many (currently, 461 defined) skeletal disorders (4).

## Mapping Rare Genetic Disorders: Early Days

It took many decades to move from the recognition of monogenic disorders to the mapping of the first gene. Initially, such genes were mapped by linkage—the co-segregation [or linkage] of a genetic region with a disease phenotype within a family. The first disease to be linked to the inheritance of any genetic marker was the dominant disorder of Huntington’s disease, initially mapped to the short arm of chromosome 4 in 1983 (5). However, it took another decade until the gene itself (*huntingtin*, located on chromosome 4p16.3) was finally determined, which effort took 58 researchers from six research groups and the participation of 75 large Venezuelan families (6). By this time, though, the first gene to be identified for any human disease had been cloned [*CYBB*, for X-linked chronic granulomatous disease (IM 300640)] (7). Linkage was often aided by recognition of chromosomal aberrations, such as translocation or uniparental disomy, in an affected individual—for example, contributing to the mapping of the gene for cystic fibrosis (8, 9). By 1995 a review article enthused about the dizzying number of genes which had been identified for human diseases—42!—marking the only time the authors have seen the phrase, “Bingo!” used in a scientific paper (10).

Gene mapping by linkage, irrespective of the chosen marker [whether chromosomal banding patterns, restriction fragment length polymorphisms, microsatellites, or single nucleotide polymorphisms (SNPs)] is critically restricted by the number of informative meioses within contributing family pedigrees. Cross-over events and recombination at meiosis incrementally limit the genetic region shared by affected individuals within the family; ergo, large multi-generational families with many affected individuals (equating to multiple meiotic events between distantly related affected individuals) represent the ideal pedigree for gene mapping *via* linkage. It would be unusual for a single pedigree to have sufficient affected individuals and sufficient informative meioses for definitive statistical evidence of linkage; thus, methods of summing genetic information from multiple families were developed. Many monogenic diseases were mapped by linkage, by 2001, 1,336 monogenic disorders [personal correspondence from Dr Victor McKusick, quoted in (11)].

There are some obvious difficulties with gene mapping by linkage. The first is that diseases with late onset or incomplete penetrance are harder to map, as correct disease attribution is more difficult. Large family pedigrees are inherently unlikely in diseases that adversely affect reproductive fitness (which includes many skeletal dysplasias, for example). The success of pooling genetic information from disparate families assumes that all affected individuals, irrespective of which family they come from, have a mutation in the same causative gene and not, for example, mutations in many different genes along a common pathway resulting in a common end phenotype. Here it is relevant to add that within any one family all affected individuals need to carry the same mutation (and, by definition, share the same haplotype of genetic markers); however, when pooling genetic information from multiple families, each family can have a different causative mutation—as long as it is in the same gene. Diseases with significant gene/environment interaction will be difficult to map—unless all family members are exposed equally to the requisite environment, essentially removing its contribution to variable affection status. Lastly, novel mutations are intractable by linkage, as by definition linkage requires the presence of a shared genetic haplotype among affected family members.

## Mapping Rare Genetic Disorders: A Complete Frameshift

In 2014, in a paper celebrating the 10^th^ anniversary of the release of the Human Genome (12) and using the example of gene mapping for fibrodysplasia ossificans progressiva (FOP; MIM 135100), we wrote that, “if massively parallel sequencing [MPS] technologies had been available when the search for the FOP gene began, the answer could have been found in 15 weeks, not 15 years.” At first glance, this statement might seem excessively hubristic even for a celebratory piece. However, to illustrate the point: at this time we had just published a review of MPS in skeletal dysplasias (13) which at the time of submission (April 2013) listed 22 skeletal dysplasias mapped using MPS with a total of 26 publications; at the time of acceptance just twelve weeks later (July 2013) ten more papers had added another six skeletal dysplasia genes to the list. The *Nosology and Classification of Genetic Skeletal Disorders: 2010 Revision* identified “456 conditions…316 [of which] were associated with mutations in one or more of 226 different genes.” (14) By the 2019 revision, pathogenic variants in 437 genes had been identified for 425 of 461 disorders now categorized (92%) (4)—*i.e.* after the decades needed to identify the first 226 genes for rare skeletal disorders, it took less than 10 years to double this number. As for skeletal dysplasias, so for many other monogenic disorders, as the mode of gene discovery rapidly transitioned from positional cloning and other traditional gene mapping methods to MPS (15, 16). Currently, the catalog Online Mendelian Inheritance in Man (https://omim.org/) lists 6,751 phenotypes for which the molecular basis is known and 4,339 genes with a phenotype-causing mutation—these numbers have increased even during the short time this paper was in review.

The key developments underpinning the extraordinary recent progress in gene mapping in rare disorders are:

- the publication of the human genome project in 2003 (17) (https://www.genome.gov/human-genome-project), providing the reference genome for comparison with sequence data.
- the development of massively parallel sequencing (MPS) technologies—both undifferentiated genome sequencing and sequencing targeted to the exome or a defined set of genes—allowing sequencing of multiple genomic regions simultaneously.
- easy accessibility of large databases of genetic variability (such as the UK10K (https://www.uk10k.org/), 1,000Genomes (https://www.internationalgenome.org/), Human Variome Project (https://www.humanvariomeproject.org/), gnomAD (https://gnomad.broadinstitute.org/) and dbSNP (https://www.ncbi.nlm.nih.gov/snp/), so that rare/novel disease-causing variants could be differentiated from more common polymorphisms within ethnically appropriate populations.
- international collaboration and cooperation, between clinicians and researchers, with interaction through platforms such as the National institute of Health Centers for Mendelian Genomics (http://mendelian.org/), Orphanet (https://www.orpha.net/consor/cgi-bin/index.php), ClinVar (https://www.ncbi.nlm.nih.gov/clinvar/), Human Gene Mutation Database (http://www.hgmd.cf.ac.uk/ac/index.php), the International Rare Diseases Research Consortium (https://irdirc.org/) and Leiden Open Variation Database (https://www.lovd.nl/), informing and encouraging collaborative new gene discovery.

Each of the above websites has detailed information about their formation and governance.

## Types of Massively Parallel Sequencing

MPS technologies can be divided into pre-defined gene panels and the more agnostic approaches of whole genome and whole exome sequencing (with abbreviations WGS and WES respectively). The authors acknowledge that, strictly speaking, WGS and WES are misnomers, as neither technology has perfect coverage of its eponymous target; however, these common abbreviations will be used in this review. There are many excellent review articles on the technical aspects of the various types of MPS (18, 19). The strengths and weakness of different MPS technologies for new gene discovery and for clinical utility are discussed below.

## Analysis of MPS Data

Human genetic variability is huge. On average, each individual harbors 3 million SNPs (5,000 private to that individual); 700,000 indels (295 private), 215 large deletions (one private), and 576 genes with either homozygous or compound heterozygous predicted loss-of-function variants (20). Sifting so much data to determine the causal variant for a disease can be, at the risk of understatement, challenging. After stringent quality control of the sequencing data, a typical common-sense and empiric approach adopted by ourselves and many others has been to filter for rare variants (with minor allele frequency thresholds informed by disease frequency and mode of inheritance) of likely deleterious effect (*e.g.*, nonsense, missense, affecting canonical splice-sites, frameshift), affecting highly evolutionarily conserved bases and predicted damaging by one or more *in silico* prediction algorithms [*e.g.*, SIFT (21), Polyphen (22), MutationTaster (23)] that segregate appropriately with disease within a family (24); or, if looking at unrelated individuals, are present in the same gene in multiple unrelated cases (25). Obviously this description is somewhat simplistic, and simply finding variants that fulfil these criteria does not prove they are disease-causing. However, these steps usually lead to a tractable list of variants that can then be assessed for functional consequence and/or compared with data from other unrelated individuals with a common phenotype.

The use of ethnically appropriate populations to determine allele frequencies for variants and inform their categorization as novel, rare, infrequent, or common, is critical. The reference data in most sequencing databases are not populated from all ethnic groups equally, with over-representation of western European Caucasian populations; more recent sequencing efforts have aimed to address this imbalance. Cohorts such as gnomAD (26) provide ethnicity-specific minor allele frequencies; but the robustness of these understandably depends on the size of the sequenced population contributing to the data.

## How Many Cases Are Needed to Map a Monogenic Disorder?

The success rate of MPS to map novel causative genes depends on the mode of inheritance of the condition. We have focused on examples drawn from skeletal dysplasias here, but the principles apply to other disease groups also.

Autosomal recessive disorders are generally easier to ‘solve’ as the list of genes with rare homozygous or compound heterozygous variants is usually relatively short. It is possible to identify the likely causative gene from initial sequencing a single affected individual (27–29)—though, as above, such evidence would need confirmation by identifying pathogenic variants in the same gene in other unrelated individuals and/or functional support.

For *de novo* dominant disorders, the causative gene may be mapped by sequencing a single affected child and parents (30) or by sequencing several unrelated probands (as few as three) and filtering the data for either a common variant shared by all affected individuals (31, 32) or with unique mutations but within a common gene (25, 33). Mapping inherited (as opposed to *de novo*) autosomal dominant diseases is more difficult due to co-inheritance of multiple unimportant variants within a family. The most parsimonious design is to sequence most distantly related affected individuals: as discussed above, these have the largest number of meioses (and, by implication, greatest number of recombination events) separating the affected cases. With *n* meioses between individuals, the chance of any given variant segregating is (½)^n^; and use of MPS data from both affected and unaffected individuals can help filter down variants according to disease status. Examples of autosomal dominant skeletal dysplasias mapped within a single family include spondylocostal dysostoses, mapped through MPS of five members of a family (three affected, two unaffected), with pathogenicity subsequently confirmed with functional data (34); and KBG syndrome [MIM 148050] (named after the initials of early affected individuals, in whom skeletal features include macrodontia, craniofacial abnormalities, and short stature), initially mapped through MPS of two affected family members and confirmed through MPS of one unrelated person (35).

Examples of X-linked skeletal dysplasias mapped by WES include the identification of mutations in *FLNA* as the cause of Terminal Osseous Dysplasia (36); and two forms of osteogenesis imperfecta, due to mutations in *PLS3* (37) and *MBTBS2* (38).

Somatic disorders can be mapped through paired analysis, with MPS of affected and unaffected tissues, subtracting the variants in the latter from the former—indeed, this approach is commonly employed in paired tumor/germline sequencing in cancer. This approach has been successful in skeletal dysplasias also—for example, identification of postzygotic somatic mutations in *PIK3CA* as the cause of Congenital Lipomatous Overgrowth with Vascular, Epidermal, and Skeletal anomalies (CLOVES), identified through WES of affected lipomatous tissue from six individuals compared with their germline DNA (39); and of *AKT1* as the cause of Proteus Syndrome (40) through WES of affected *vs.* unaffected tissue biopsies in 29 individuals. Depth of coverage will critically affect the ability to detect mosaicism, in that the allelic ‘mix’ in somatic disorders will vary both between individuals and between different tissues within an individual. The acceptable depth of MPS for calling germline heterozygous carriage of a variant is relatively modest: 10× is usually regarded as sufficient to ‘call’ a heterozygous variant and 15× for a homozygous variant (41); at these depths of coverage WES would be unlikely to detect low level mosaicism.

## Strengths and Weakness of Different MPS Technologies for New Gene Discovery and for Clinical Utility

In keeping with early predictions that 85% of Mendelian disorders would arise from coding mutations (42) and with the logic inherent in Sutton’s law (*viz*., that one robs banks because that’s where the money is), it is neither surprising that WES has been the most frequently employed modality to map novel genes, nor how successful this approach has been. Most of the examples provided above used WES as their mode of gene discovery, and the figure given above may well prove an underestimate. WES is not simply much cheaper than WGS for a given coverage: the large databases detailing exonic variation that informs analysis of WES data do not as yet exist for the whole genome (though this is rapidly changing with initiatives such as the UK Biobank 500K Sequencing Project and gnomAD), and proving causality for non-coding variants is difficult.

WES has proven similarly fruitful in diagnostic yield when translated from the research setting to clinical delivery [recently reviewed extensively (43)] with high diagnostic rates reported in both developed and developing countries (44), including sequencing in consanguineous families (44–46) and singleton sequencing (47) [noting that yield is approximately two-fold higher when sequencing parent–child trios compared with singletons (43)]. WES may also lead to a revision of a diagnosis—which may be confronting to both patient and clinician (discussed further below) but hopefully direct more appropriate clinical care (45, 47). A recent study reporting 155 novel causal genes identified during clinical sequencing (WES) in a consanguineous cohort comprising 2,200 families highlighted not only the use of WES for diagnostic purposes but also the benefits of these data in completing the virtuous circle of clinical discovery and clinical delivery, through feedback of these data for ongoing research and gene discovery (44). However, WES is not ideal for detection of copy number variation (48) including detection of large indels.

Very few monogenic disorders due to non-coding/splice-site variants have been identified to date (49). Ironically, a notable exception to this is the skeletal disorder of van Buchem’s disease, a high bone mass disorder due to a 52 kb deletion downstream of *SOST* (50), though this disorder was not identified through MPS approaches. Thus, the usefulness of WGS in gene discovery in monogenic disorders, compared to WES, has not yet been established. Certainly WGS captures the exome more evenly (as well, obviously, as the genome) than does WES. WGS is also superior for the detection of large (>50 bp) indels, copy number variation, and chromosomal rearrangements. The higher costs of WGS and analysis are rapidly falling (51); and thus choosing between sequencing technologies from a purely fiscal perspective may soon be redundant. Nonetheless, to date WGS has not demonstrated superiority to WES in diagnostic utility (43); and the extent to which WGS may ultimately provide a diagnosis in cases for which WES has failed to identify a cause is not known.

By definition, a targeted panel approach cannot be used for new gene discovery, as such panels consist of already identified genes. Nonetheless, panel sequencing has an established place within clinical delivery as a cheap, sensitive, and specific means of sequencing known disease genes, with excellent coverage due to the limited targeted region, and minimization of some of the concerns raised with the agnostic approaches such as WES or WGS such as incidental or secondary findings (discussed below). However, the first-line use of WES, rather than panel approaches—even when up to three panels were chosen by expert clinical geneticists—shortens the diagnostic odyssey and is more cost-effective (52).

Considering clinical utility of MPS technologies for bone diseases specifically, both WES (53) and panel sequencing (54) approaches have been reported. There are no inherently unique issues pertaining to clinical use of MPS in skeletal diseases compared to other disorders.

## Incorrect Attribution of Pathogenicity

A variant is only rare when considered against the population; within a family, a rare variant is not rare—it has a 50% chance of transmission from a parent to a child; similarly siblings will share a variant identical-by-descent on average 50%. It is extremely easy to be tempted into attributing causality to a rare variant that segregates within a small family just because it is rare [discussed in depth in (55) and (56)]. However, *a priori* one can predict the chance that any particular variant will segregate with disease within a family according to the number of meiosis between affected individuals and within a small family that probability may be higher than the typical threshold for declaring scientific significance (*i.e.* p < 0.05). Unsurprisingly, in a review article on this topic, MacArthur et al. wrote that of “406 published severe disease mutations….122 (27%) were either common polymorphisms or lacked direct evidence for pathogenicity” (56).

Efforts to refine criteria for attributing pathogenicity to an identified variant led to the publication of guidelines for classifying the likely pathogenicity of identified variants (*e.g.* ‘pathogenic’, ‘likely pathogenic’, ‘variants of uncertain significance’, *etc.*) according to the strength of evidence (57). These guidelines recommend using multiple criteria and resources to guide classification of an individual variant into a particular category, including population, disease-specific, and sequence databases, the published literature, the type of variant (nonsense, frameshift, initiation codon, canonical splice-sites, large deletions, *etc.*), and *in silico* prediction algorithms. However, considering the evidential basis even within these criteria demonstrates the imperfections. There are multiple *in silico* prediction methods, each with differing criteria (gene-level, variant level, evolutionary conservation, amino acid change, *etc.*) trained on varying datasets—not surprisingly, they vary in performance [recently discussed and compared in (58)]. Replication—observing the same mutation with the same phenotype in an unrelated family—depends on cooperation and collaboration of researchers, and for rare diseases this needs to happen at an international level—which depends on clinical networks. Clinical variation databases (*e.g.*, ClinVar, Online Mendelian Inheritance in Man, Leiden Open Variation Database, Human Gene Mutation Database) rely on curation expertise. Altruism is a key component for the success of any database [including PubMed (https://pubmed.ncbi.nlm.nih.gov/)]—however, clinical reporting of affected cases requires awareness, motivation, confidence, and time. Thus, functional studies, in either *in vitro* or *in vivo* models, are often necessary for definitive classification. To this end, CRISPR technology (for which discoverers Emmanuelle Charpentier and Jennifer Doudna were recently awarded the 2020 Nobel Prize for Chemistry) has proven a boon.

## Ethical, Legal, and Social Implications in Massively Parallel Sequencing Technologies

Whatever type of genetic testing is performed—whether MPS or other technologies—pre-test discussion is crucial to ensure the individual is aware of all possible outcomes and their implications, both for the individual personally and for their family members. Some considerations are universally long-recognized risks associated with any type of genetic test (discussed further below). However, MPS can add to the magnitude of risk and/or complexity of results, as well as generating issues specific to the technology, such as secondary findings.

### Relationship Misattribution

For decades, clinical genetics professionals have faced the challenge of misattributed relationships identified through genetic testing, especially non-paternity. Most genetics clinicians only disclose this information when clinically necessary (59, 60). Moreover, in accordance with the Institute of Medicine Guidelines (61), non-paternity results [estimated to be present in up to 30.0% of livebirths (62)] are only disclosed to the mother alone. With genetic tests ordered in many more settings and much more frequently, the risk of uncovering misattributed relationships is extremely likely to increase (63). In addition, misattributed relationship results generated by single-gene tests are often associated with some degree of uncertainty, which allows for some degree of clinical discretion. In contrast, the simultaneous identification of both common and rare variants inherent in any MPS technology generates unequivocal results (63).

### Disclosure of Genetic Status Through Relationships With Other Family Members

The shared nature of genetic material means that a positive test result in one individual can reveal the genetic status of other family members by inference. This may be due to their affection status (*e.g.*, a *BRCA1* result in a woman with breast cancer implies mutation carriage in her mother with ovarian cancer) or the nature of inheritance (*e.g.*, the obligate carrier status of parents whose child is diagnosed with a recessive condition).

### Unexpected Results Related to the Disease in Question

Genetic tests have the potential to yield information about the future health of an individual, who may be clinically unaffected at the time of testing. In single gene testing for carrier status, careful predisposition testing protocols were developed, particularly for neurodegenerative (64) and cancer susceptibility syndromes (65), to ensure individuals were prepared for the clinical, psychological, and logistical sequelae of learning such information. Preparing an individual for testing by MPS is challenging from a counseling perspective, if only for the large number of genes being tested simultaneously. However, more subtle issues may arise—for example, a causal gene may be identified that differs from the expected gene (66); and the results may confer an increased risk for conditions not previously described in the family or not previously recognized to be significant (*e.g.*, a *TP53* mutation in a family with a strong history of breast cancer).

### Variants of Uncertain Significance

Variants of uncertain significance (VUSs) are variants for which there is insufficient evidence to classify them as benign or pathogenic. As additional information becomes available over time, they are sometimes re-classified as pathogenic/likely pathogenic or, more commonly, benign/likely benign (67–69). VUSs have been a long-standing challenge in genetic testing for hereditary cancer generally (68) and *BRCA1/2* specifically (67). The larger the number of genes interrogated, the higher the probability of generating a VUS: 36 and 73% in multigene panels (70) and exome sequencing (71) respectively. A recent systematic review found VUSs are associated with genetic test-specific concern and affects clinical management (72).

### Incidental or Secondary Findings

Incidental or secondary findings are genetic test results unrelated to the primary condition. Incidental findings are generally regarded to be inadvertent or accidental discoveries emerging during data analysis. In contrast, secondary findings emerge from the deliberate interrogation of ‘actionable’ genes in individuals undergoing WES or WGS, with the goal of prevention or early detection of treatable conditions. To overcome the challenge of terminology, these are cumulatively referred to as incidental and secondary findings (ISFs) (73).

In 2013, the American College of Medical Genetics published guidelines recommending that all individuals having WES/WGS have automatic analysis of 56 actionable genes, associated with 24 hereditary cardiac or cancer predisposition syndromes (74). Among other statements, the guidelines stated that neither patient age nor patient preferences should be taken into account because this would be “logistically challenging” for laboratories (74). The paper stimulated multiple articles in response. Concerns raised included the lack of scientific evidence to support screening of all 56 genes, with insufficient information about phenotype and penetrance (75, 76). The potential for large numbers of VUSs was also recognized as was the challenge of interpreting variants in ethnic minorities (77). The potential for iatrogenic harm or false reassurance was raised. Multiple papers stated that the guidelines disregarded individual autonomy (78, 79) and contravened the ACMG’s own guidelines on genetic testing in children (75)—with overlapping concerns of lack of informed consent (75). The second version of the guidelines removed the wording around any obligation to interrogate these genes whenever WES/WGS and acknowledged that all patients should have the right to opt out—and modified the medically actionable genes to a slightly different list with the overall number increased to 59 (80). At present, some laboratories offer secondary screening of the ACMG 59^TM^ (81); however, the extent to which it has been adopted by clinical laboratories world-wide is unclear. Additionally, there is ongoing debate about whether the ACMG 59™ should be offered and reported in the prenatal period (82). The ACMG Board of Directors recently released a policy statement stating that they do not support the use of ACMG 59™ as a screening tool in the general population (83).

### Genetic Discrimination

Fear of genetic discrimination, particularly as it pertains to insurance underwriting, is a deterrent in the pursuit of clinically indicated genetic testing (84–86). Several papers suggest these fears are not ill-founded, with incidences of proven or alleged genetic discrimination reported in carriers of recessive conditions (87–89) and—perhaps surprisingly—individuals receiving a negative (*i.e.* good news) result in predictive testing for familial mutations (88, 89) and healthy carriers of dominant variants who pursued surgical/medical interventions and/or screening to mitigate their risk (89–96). Policies and legislation have been introduced in many countries (including the UK, US, Canada, Australia, and European countries) to limit or prohibit the use of genetic test results in insurance underwriting (97), but initial studies suggest that awareness of such legislation among non-genetics clinicians (98) and members of the public (99, 100) is low. For example, a UK study found that *BRCA1/2* carriers had difficulty obtaining insurance even after the introduction of the Concordat and Moratorium on Genetics and Insurance (95).

### Equity

Personalized (or precision) medicine aims to improve care by customizing management to the individual and the profile of their disease. Genetic testing is an integral component of personalized medicine and encompasses a gamut of approaches, from tumor sequencing [*e.g.*, improving survival through targeted chemotherapy (101)] to common variant genotyping [*e.g.*, use of polygenic risk scores, usually determined through microarray technology (102)] to rare variant detection by MPS technologies (as discussed above). Access to genetic services is limited by racial, ethnic, and social factors; and disproportionate access has potential to widen, rather than reduce, health disparities both within developed countries (103, 104) and between developed and developing countries (105) [though here we would highlight increasing use of MPS technologies clinically in communities with higher rates of intrafamilial marriage (44–46)].

## Final Thoughts: Access to Sequencing and Future Directions

In 2016, one of the current authors wrote “Conventional sequencing is commercially available for a finite number of mutations in clear-cut monogenic diseases—but these conditions represent a minority of genetic disorders. In Australia, genetic testing is available for 597 genes which cause <500 different syndromes and conditions, a small subset of the ~5,000” [McInerney-Leo, PhD thesis; data drawn from the Royal College of Pathologists, Australia, accessed 2016 (http://genetictesting.rcpa.edu.au)]. Just four years later, the situation is very different, with both public and private access to testing for multiple conditions in Australia and in many countries around the world. A recent review article led with an arresting title of *A Diagnosis for All Rare Genetic Diseases: the Horizon and the Next Frontiers*, (49) and presented a vision that all families with a rare genetic disorder would ultimately receive a genetic diagnosis through sequencing technologies and novel data analyses approaches. This aim is not only exciting but with ongoing international cooperation and collaboration—even mid-coronavirus—it also seems achievable (49).

## Author Contributions

Both AM-L and ED prepared and reviewed this manuscript. All authors contributed to the article and approved the submitted version.

## Funding

AM-L is funded by a National Health and Medical Research Council (NHMRC) Early Career Fellowship (ID 1158111).

## Conflict of Interest

The authors declare that the research was conducted in the absence of any commercial or financial relationships that could be construed as a potential conflict of interest.
